# Autonomic Neural Circuit and Intervention for Comorbidity Anxiety and Cardiovascular Disease

**DOI:** 10.3389/fphys.2022.852891

**Published:** 2022-04-27

**Authors:** Xuanzhao Chen, Li Xu, Zeyan Li

**Affiliations:** ^1^ The Center of Pathological Diagnosis and Research, Affiliated Hospital of Guangdong Medical University, Zhanjiang, China; ^2^ Department of Rheumatology and Immunology, General Hospital of Central Theater Command, Wuhan, China

**Keywords:** anxiety, nuclei, cardiovascular system, autonomic nervous system, central nervous system

## Abstract

Anxiety disorder is a prevalent psychiatric disease and imposes a significant influence on cardiovascular disease (CVD). Numerous evidence support that anxiety contributes to the onset and progression of various CVDs through different physiological and behavioral mechanisms. However, the exact role of nuclei and the association between the neural circuit and anxiety disorder in CVD remains unknown. Several anxiety-related nuclei, including that of the amygdala, hippocampus, bed nucleus of stria terminalis, and medial prefrontal cortex, along with the relevant neural circuit are crucial in CVD. A strong connection between these nuclei and the autonomic nervous system has been proven. Therefore, anxiety may influence CVD through these autonomic neural circuits consisting of anxiety-related nuclei and the autonomic nervous system. Neuromodulation, which can offer targeted intervention on these nuclei, may promote the development of treatment for comorbidities of CVD and anxiety disorders. The present review focuses on the association between anxiety-relevant nuclei and CVD, as well as discusses several non-invasive neuromodulations which may treat anxiety and CVD.

## Introduction

Cardiovascular disease (CVD) is a major contributor to disability and the leading cause of death worldwide (Roth et al., 2020). While effective drugs are widely used for CVD in clinical practice, significant gaps still exist in the treatment of CVDs (Flora and Nayak, 2019). Some psychological factors, such as anxiety, can influence the onset and progression of CVDs. An increasing number of studies have discussed the impact of clinically relevant anxiety not only on CVD-related mortality but also on all-cause mortality (Meier et al., 2016). However, it is inconclusive whether treatment for anxiety and depression can prevent CVDs and improve the outcome of CVDs (Piepoli et al., 20162016). Although the association between CVDs and anxiety disorder may be attributed to numerous biological and behavioral mechanisms, the role of anxiety-related nuclei in CVDs remains unknown (Celano et al., 2016). Several nuclei have been identified as the key loci controlling anxiety, including that of the amygdala, hippocampus (HC), bed nucleus of stria terminalis (BNST), and medial prefrontal cortex (mPFC). Decoding the microcircuits relevant to these nuclei, as well as those of regional microcircuits, also helps to improve our understanding of anxiety (Calhoon and Tye, 2015). These nuclei possess intimate connections with brain regions, such as the hypothalamus and brainstem, which are involved in the autonomic nervous system (ANS) (Linsambarth et al., 2017). Nevertheless, the role of these nuclei and neural circuits in linking anxiety to CVD remains to be elucidated. Recently, a series of studies have demonstrated the influence of these nuclei and circuits on cardiovascular response and disease (Granjeiro et al., 2012; Scopinho et al., 2013; Tawakol et al., 2017; Moazzami et al., 2020). The current review will briefly discuss the relationship between anxiety and CVDs and introduce several treatment strategies which potentially alleviate CVD comorbidity with anxiety through targeting subregions of the central nervous system (CNS).

## Anxiety in Patients With CVDs

Anxiety is characterized by uncertainty, apprehension, and transient fear for the future, with the frequency and intensity varying between different individuals. Distinctions between normative anxiety and anxiety disorders require a clinical judgment of the duration, severity, persistence, and degree of distress and impairment (Penninx et al., 2021). Anxiety disorders are common in patients with CVDs with greater prevalence than in the general population (Piña et al., 2018). Some meta-analyses have suggested that anxiety disorders might contribute to the onset and development of CVDs (Strik et al., 2003; Batelaan et al., 2016). In addition, a series of studies not only considered the impact of anxiety on CVD-relevant mortality but also on all-cause mortality (Chesney et al., 2014; Meier et al., 2016; Pratt et al., 2016; Sokoreli et al., 2016). In this regard, the following sections will focus on the relationship between anxiety disorder and different CVDs.

### Anxiety and Heart Failure

Anxiety disorder is prevalent in patients with heart failure (HF). The result from a meta-analysis of 26,266 patients with HF indicated that almost 30% of patients with HF reached a clinically significant degree of anxiety (Easton et al., 2016). Furthermore, anxiety may affect the mortality of patients with HF. Existing evidence has highlighted the significance of determining anxiety disorders in patients with HF to improve clinical outcomes. First, anxiety is related to higher rates of mortality and poor cardiovascular health in patients with coronary artery disease, which often co-exist with HF (Roest et al., 2010a; Roest et al., 2012; Celano et al., 2015). Moreover, in patients with or without CVD, different types of anxiety disorders such as panic disorders, generalized anxiety disorders, and post-traumatic stress disorder (PTSD) are related to poor cardiovascular outcomes (Smoller et al., 2007; Boscarino, 2008; Walters et al., 2008; Ahmadi et al., 2011; Roest et al., 2012; Edmondson and Cohen, 2013). In addition, in patients with HF and co-occurring depressive symptoms, the existence of comorbid anxiety enhances the risk of poor outcomes, including mortality and rehospitalizations (Suzuki et al., 2014; Alhurani et al., 2015).

Despite the evidence suggesting that anxiety is common in patients with HF, research that compared depression as comorbidity found less prevalence, trait, and influence of anxiety among these patients (MacMahon and Lip, 2002; Merikangas et al., 2003; Konstam et al., 2005). Therefore, patients with HF may benefit from recognition and treatment of anxiety. However, accurate diagnosis of anxiety with a physical disease can be challenging owing to both emotional and physical shared symptoms such as chest pain, fatigue, palpitations, and breathlessness (Singleton et al., 2003; Easton, 2013). To enhance the diagnosis of anxiety and depressive disorders in patients with HF, the American Heart Association has suggested screening for common psychiatric diseases (Kroenke et al., 2001; Kroenke et al., 2003).

Despite the benefits of screening for anxiety disorders in patients with HF, the practical details of screening are less clear. For example, anxiety symptoms may be apparent following an acute CVD, but these symptoms may dissipate after recovery from events. Accordingly, if the first diagnosis of anxiety is made during admission, it is advisable to defer the final diagnosis of anxiety to a stage of clinical stability.

### Anxiety and Atrial Fibrillation

Atrial fibrillation (AF), a kind of atrial tachyarrhythmia, is the most common type of durative tachyarrhythmia in clinical practice (Michaud et al., 2021). According to statistics from the Framingham Heart Study, the incidence of AF developed in 37% of patients over 55 years old (January et al., 20142014; Staerk et al., 2018). Several clinical studies have demonstrated a strong association between the initiation or recurrence of AF and anxiety symptoms. For instance, a 10-year observational trial found that the incidence of AF can be influenced by anxiety (Eaker et al., 2005). Furthermore, it was found that after cardiac surgery, anxiety symptoms can increase the occurrence of AF, whereas the correlation between AF and anxiety could be reduced through beta-blockers (Tully et al., 2011; Tarsitani et al., 2012). In addition, Pitsavos et al. found that anxiety is associated with abnormal coagulation and systemic inflammation—probable contributors to increased cardiovascular events (Pitsavos et al., 2006). Not only did anxiety influence the onset and progression of AF but may also lead to AF recurrence after standard treatment for AF. Yu et al. found that after taking circumferential pulmonary vein ablation, patients with anxiety and AF were at higher risk of AF recurrence (Yu et al., 2012). In a clinical study, researchers found that paroxetine, an anti-depressant drug, can further decrease frequency of arrhythmia events in patients with multidrug-resistant paroxysmal AF, probably through inhibiting the vasovagal reflex and regulating vagal tone (Shirayama et al., 2006). However, no evidence has demonstrated anti-anxiety drugs lower the incidence of AF.

According to the clinical studies mentioned above, several limitations for the research into the relationship between anxiety and AF should be noted, such as using different questionnaires with diverse validity and reliability, small sample size, and short follow-up period. Therefore, future research should address these disadvantages. Furthermore, large prospective studies are essential to evaluate the benefits of routine assessments of anxiety, and the usefulness of anti-anxiety drugs in the prevention and treatment of patients with AF and anxiety.

### Anxiety and Coronary Heart Disease

A meta-analysis of 20 studies (*N* = 249,846) determined the association between anxiety and incident coronary artery disease (CHD) and found that initially, healthy participants with anxiety were at elevated risk for incident CHD and cardiac death, independent of health behaviors, biological risk factors, and demographic variables (Roest et al., 2010b). Another meta-analysis of 46 studies indicated that anxiety was related to a 35% greater risk of HF, 71% greater risk of stroke, and 41% greater risk of cardiovascular mortality and CHD (Emdin et al., 2016). Furthermore, anxiety has been considered as a potential risk factor for myocardial infarction (MI) in men (Shen et al., 2008). In this regard, the manifestation of either anxiety or depression or mixed manifestation contributes to a 20%–30% elevation in the risk of MI (Ouakinin, 2016). Moreover, patients with over two types of psychological disorders had a 50% higher risk of MI following the next 10 years (Ouakinin, 2016). High levels of anxiety symptoms before MI can also exacerbate long-term outcomes in the elderly (Smeijers et al., 2017). In addition, Roest et al. suggested that anxiety after MI may be a prognostic factor that increases the risk of the worse outcome by 36%. On the other hand, the elevation of anxiety after MI might be temporary but can persist for the first two years after MI (Bjerkeset et al., 2005). Post-MI anxiety disorders are related to a higher risk of recurrent MI (Feng et al., 2016). However, the degree of anxiety and clinical events (including all-cause death and MI) can be significantly alleviated in patients after stress management training (Blumenthal et al., 2016). Anxiety does not only influence initiation, development, and prognosis of CHD but can impact the treatment of CHD. Co-existing symptoms of depression and anxiety can be significant predictors of worse outcome after percutaneous coronary intervention (PCI) (Pedersen et al., 2006; van den Berge et al., 2015; van Dijk et al., 2016). Furthermore, patients experiencing anxiety before coronary artery bypass graft surgery (CABG) had higher risk of mortality (Tully et al., 2008; Tully et al., 2015). Post-CABG anxiety is also associated with the higher risk of acute MI, recurrent hospitalizations, and mortality (Rosenbloom et al., 2009; Poole et al., 2017).

### Anxiety and Hypertension

Numerous evidence from clinical trials has demonstrated the relationship between anxiety and hypertension. On the one hand, compared with patients without an anxiety disorder, baseline anxiety was related to a higher rate of developing incident hypertension (odds ratio [OR] 4.24; 95% CI 1.29–14.01) (Bacon et al., 2014). Although adjusting for age, country, gender, and other psychosocial disorders, divergent types of anxiety disorders are associated with the development of incident hypertension (Stein et al., 2014). Another larger prospective cohort study (2005–2015) included 524,952 patients who suggested that the baseline diagnosis of anxiety can increase the risk of incident hypertension (hazard ratio HR 1.09; 95% CI 1.05–1.14, *p* < 0.001) (Pérez-Piñar et al., 2016). On the other hand, a positive, bidirectional link may exist between prevalent hypertension and prevalent anxiety, i.e., patients with anxiety were more likely to have hypertension and vice versa (Player and Peterson, 2011). The World Mental Health Survey, initiating 18 cross-sectional studies in 17 countries among the general public, has found that the adjusted OR for comorbid hypertension and anxiety was 1.7 (95% CI 1.5–1.9, *p* < 0.05) (Scott et al., 2007). A recent cross-sectional medical record analysis assessed the prevalent comorbid hypertension and mental disease, including anxiety (*n* = 2,058,408). Overall, ambulatory and residence patients with hypertension were more likely to possess medical record diagnoses of anxiety (Sandström et al., 2016). Furthermore, some studies have suggested that symptoms of anxiety may be associated with hypertension and the change of BP (Hildrum et al., 2008; Wu et al., 2014). Blood pressure variability (BPV) represents the size and patterns of BP variations from seconds to years and is considered a marker of ANS regulation and an independent risk indicator of cardiovascular complications (Wei et al., 2018). And BPV is considered as a predictor of initiation, progression, and severity of organ damage caused by hypertension (collectively a marker of ANS dysregulation), as well associated with anxiety disorder (Irigoyen et al., 2016).Several cross-sectional studies found that higher anxiety scores are related to lower heart rate variability (HRV) and higher BPV indicating ANS imbalance towards sympathetic hyperactivity (Piccirillo et al., 1997; Tully and Tzourio, 2017). However, the recognition and treatment for anxiety disorders and hypertension remain insufficient (Johnson et al., 2014; Bandelow and Michaelis, 2015). Therefore, it is of great significance to improve our comprehension of comorbid anxiety and hypertension.

## Anxiety-Related Nuclei in CVD and Cardiovascular Response to Stress

Several studies have demonstrated a correlation between elevated HR and BP reactions, as well as enhanced activation in central neural limbic and brainstem regions in response to mental stress among healthy individuals (Gianaros and Sheu, 2009; Gianaros et al., 2012). Patients with CVD present structural and functional changes in neural networks including the frontoparietal, limbic, and brainstem regions (Gianaros et al., 2009a; Gianaros and Sheu, 2009; Jennings and Zanstra, 2009). These studies have suggested that abnormalities in brain nuclei of groups at high risk for developing CVD are related to exaggerated cardiovascular response to stress, which may contribute to the initiation and progression of CVD. Accordingly**,** such frequent excessive cardiovascular responses caused by anxiety may also prompt structural changes in the cardiovascular system and ultimately lead to the development of acute and chronic CVD. An important foundational study has identified several key components controlling anxiety, including the amygdala, BNST, mPFC, and HC (Calhoon and Tye, 2015). The following section focuses on the role of these nuclei in cardiovascular response and disease in healthy individuals and patients.

### Role of the Amygdala in CVD

Existing evidence from human studies indicates the importance of the amygdala to anxiety (Etkin and Wager, 2007; Grupe and Nitschke, 2013). Early animal studies related to fear conditioning emphasized the vital roles of the central nucleus of the amygdala (CeA) and the basolateral amygdala (BLA) in anxiety. The BLA receives sensory information from stress and excites the CeA through its projections. Subsequently, the amygdala contributes to defensive responses through efferent projections to different regions, including the stria terminalis, hippocampus, ventral striatum, orbitofrontal cortex, periaqueductal gray (PAG), and hypothalamus (LeDoux, 2000). Recently, several clinical studies explored the relationship between the amygdala and cardiovascular response and disease (Gianaros et al., 2008; Gianaros et al., 2009a; Gianaros et al., 2009b; Tawakol et al., 2017; Tawakol et al., 2019; Goyal et al., 2020; Osborne et al., 2020).

Despite lacking updated human cerebrum imaging techniques to confirm the precise construction and function of diverse amygdalas, Gianaros et al. indicated the activity of the amygdala can be used to reflect the alterations of BP to stress and predict the risk of preclinical atherosclerosis (Gianaros et al., 2008; Gianaros et al., 2009a; Gianaros et al., 2009b). The first study, linking focal brain activity to CVD events has demonstrated that amygdala activity can be used as an independent and robust predictor of CVD events. The degree of amygdala activation can be used as a marker to foresee the occurrence of heart attacks and strokes as it was positively associated with hazards for stroke and heart attack. The study indicated that the activity of anxiety-related nuclei may be the potential neural substrate for cardiovascular risk (Tawakol et al., 2017). Several other clinical observational studies have also demonstrated different types of stress, including noise, psoriasis, and socioeconomic status can pose a significant impact on amygdala activity and may subsequently induce arterial inflammation contributing to CVD (Tawakol et al., 2019; Goyal et al., 2020; Osborne et al., 2020).

Identifying that the degree of amygdala activation in humans can be used as a predictor of cardiovascular responses, such as alterations of BP and HR, and CVD outcome is a great breakthrough. However, elucidating the intricate intra-amygdala interactions, which may act as a mechanism related to CVD, require further investigations.

### Role of the BNST in Cardiovascular Response to Stress

The BNST, which is considered as part of the extended amygdala, has similar cytoarchitecture and strong contact with the amygdala (McDonald, 2003; Price, 2003). Sustained anxiety responses require the recruitment of the BNST (Davis et al., 2010), which emerges partly as a result of direct innervation by BLA afferents. A recent study using optogenetic targeting of different BNST subregions and output pathways found opposing roles for the oval BNST (ovBNST) and anterodorsal BNST (adBNST) in anxiety (Kim et al., 2013) Existing evidence has suggested the role of BNST in cardiovascular response and its potential mechanism.

Human functional magnetic resonance imaging (fMRI) studies have reported BNST activation in response to threat anticipation (Straube et al., 2007; Mobbs et al., 2010; Somerville et al., 2010; Alvarez et al., 2011; Grupe et al., 2013; McMenamin et al., 2014; Klumpers et al., 2017). Among these studies, recent research (*n* = 178) found that the BNST was implicated in defensive response during uncertain threat anticipation (Klumpers et al., 2017). The study demonstrated that stress anticipation and stress confrontation, respectively, evoke bradycardic and tachycardic responses with neural activity shifted from a region anatomically consistent with the BNST toward the amygdala. This reinforced the previous view that BNST is implicated in defensive responding during uncertain threat anticipation, whereas the amygdala may drive response upon more acute danger (Klumpers et al., 2017). Furthermore, another research explored the association between the activity of the BNST and peripheral ANS (Somerville et al., 2010). In this study, the participants with greater anxiety about the threat proximity showed more prominent activity of the BNST and sympathetic nervous system which displayed a significant increase in HR and skin conductance (an activity indicator of the sympathetic nervous system).

According to these results, it may be assumed that the BNST, as an anxiety-related nuclei location, may play an important role in the initiation and progression of CVD as part of its regulation of cardiovascular responses to different stress. However, the exact relationship between the BNST and CVD is unknown. Therefore, clinical and basic research is needed to disentangle the potential link between these nuclei and CVD in the future.

### Role of the mPFC in CVD and Cardiovascular Response to Stress

The mutual relationship between the medial prefrontal cortex (mPFC) and the amygdala has been comprehensively investigated in anxiety disorders in both humans and rodents (Ochsner et al., 2002; Kim et al., 2011). The mPFC, a neocortical region in the central neocortical structure with substantial excitatory pyramidal neurons and different kinds of inhibitory interneurons, consists of six layers in humans (I–VI) and only five organized stratums in rodents (Markram et al., 2004). The mPFC in rodents can be divided into two parts, including the prelimbic (PL) and infralimbic (IL) cortex. These subregions in mPFC accept projections from the thalamic nuclei, BLA, and HC, which then project to the BLA and striatum (Groenewegen et al., 1997). Several studies have demonstrated the relationship between the activity of the mPFC and CVDs, as well as in response to stress in humans.

The important role of the interaction between the mPFC and limbic regions in HR regulation has been corroborated using MRI and resting-state functional connectivity (RSFC) (Sakaki et al., 2016; Kumral et al., 2019). In a clinical observation trial, researchers found that an increased RSFC between the ventro-mPFC (vmPFC) and the anterior insula was associated with slower HR (de la Cruz et al., 2019). In a recent publication, researchers also demonstrated the role of the vmPFC in regulating the cardiac autonomic function. They found that temporal changes in HRV were correlated with dynamic changes in prefrontal connectivity (Schumann et al., 20202008). HRV indicates the degree of continuous change in heart rate through the analysis of a series of heartbeat intervals considered as a clinical marker for the state of the ANS. This index can be generated through several calculations, including a time-domain method, geometric method, and frequency domain methods (Author Anonyms, 1996). In addition, the results from other clinical studies indicated that lesions of the cerebral cortex, especially in the mPFC, were related to the exaggeration of HR response during mental stress (Buchanan et al., 2010). Furthermore, the activity of mPFC to stress may be linked to CVD. Researchers have found greater stress activation of the rostro-mPFC (rmPFC) in patients with CHD exposed to early traumatic events, as well as those who exhibit high-stress reactivity with peripheral vasoconstriction (Shah et al., 2019; Wittbrodt et al., 2019). A recent publication (including 148 subjects) has found that higher rmPFC stress reactivity was independently associated with higher IL-6 and a lower high-frequency power spectrum index of HRV with stress. During a median follow-up of 3 years, 34 subjects (21.3%) experienced major adverse cardiovascular events (MACE). Each 1SD (standard deviation of relative cerebral blood flow measured by high resolution-positron emission tomography) increase in rmPFC activation with mental stress was associated with a 21% increased risk of MACE (HR 1.21, 95% CI 1.08–1.37) (Moazzami et al., 2020).

The results mentioned above have indicated that the mPFC may be important to CVD, which is a potential target for anxiety comorbidities in CVD. Therefore, it is of significance to explore the mechanism of mPFC resulting in CVD.

### Role of the HC in CVD

Correlational and epidemiological studies have implicated the HC in human psychological disorders, including anxiety (McNaughton, 1997). Behavioral, anatomical, and gene expression studies have suggested that the HC in rats comprises two distinct subregions compared to the posterior HC in primates, and the ventromedial hippocampus (VH) is similar to the anterior HC in primates (Risold and Swanson, 1996; Fanselow and Dong, 2010). Despite the comprehensive acknowledgment of the intimate correlation between the activity of HC with emotional and mnemonic function, few studies have focused on the relationship between the role of the HC and cardiovascular responses to stress (Ai et al., 2015).

Nevertheless, a recent series of studies have demonstrated the relationship between the HC and CVD, as well as the involvement of the HC in cardiovascular system regulation. For instance, patients with temporal lobe epilepsy and hippocampal sclerosis demonstrated cardiovascular autonomic dysfunction (Ansakorpi et al., 2004; Koseoglu et al., 2009). In a most recent study of 80 patients, Mueller et al. (2020) investigated a potential correlation between HF biomarkers and the brain gray matter density (GMD) obtained by MRI. They observed a diminished GMD was associated with decreased ejection fraction and increased NT-proBNP in various brain regions including the whole frontomedian cortex as well as the HC and precuneus. In addition, a smaller observation trial found that patients with HF exhibited smaller hippocampal volumes than controls (right: 3,060 ± 146 vs. 3,478 ± 94 mm³; *p* = 0.02; left: 3,021 ± 145 vs. 3,352 ± 98 mm³; *p* = 0.06) (Woo et al., 2015). Moreover, a largescale study intending to investigate the relationship between hypertension and memory indicated a correlation existed between the history of hypertension and both lessor functional connectivity of the HC and lessor prospective memory score (Feng et al., 2020). Furthermore, hippocampal atrophy is a significant and independent predictor of poor prognosis in patients with chronic HF and can aid the risk stratification of these patients (Niizeki et al., 2019). Therefore, the altered structure of the HC may influence the regulation of cardiovascular function and CVDs.

Although evidence supporting the effect of HC on CVD is scarce, the existing results indicate that anxiety-related nuclei may play an important role in CVD.

## Link Between Anxiety-Related Nuclei and Cardiac ANS

The central neural autonomic network (also denoted the ANS), composed of several brain structure interconnections, was confirmed by observation of the human CNS via human brain imaging, and by investigation of rodent CNS via tract-tracing and electrical stimulation (Verberne and Owens, 1998; Saper, 2002). In the CNS, the dorsal motor nucleus of the vagus (DMV) and nucleus ambiguus (NA) are the primary sites of preganglionic parasympathetic neurons that regulate the heart (Ulrich-Lai and Herman, 2009). Adversely, the intermediolateral cell column (IML), which is in the thoracolumbar range of the medulla spinalis, projects preganglionic sympathetic neurons to regulate the heart (Kandel et al., 2000; Bear et al., 2007).

Several brain nuclei, including the hypothalamus, brainstem, etc., directly project to the DMV—particularly the subarea of the hypothalamus, including the lateral hypothalamic area (LHA) (Hahn and Swanson, 2010), medial preoptic area (mPOA) (Chiba and Murata, 1985), and paraventricular hypothalamus (PVH) (Swanson and Kuypers, 1980). The projection from the brainstem to the DMV mainly originates from A1 cell groups of the ventrolateral medulla (VLM) (Sawchenko and Swanson, 1981), nucleus of the solitary tract (NTS) (Davis et al., 2004), and locus coeruleus (LC) (Ter Horst et al., 1991). In addition, the reticular nuclei and vagal complex, which are both important nuclei locations in the brainstem, display abundant interaction with each other. The projection from the NTS (Sawchenko and Swanson, 1982) and the parabrachial nuclei (PB) (Herbert et al., 1990) can reach NA and DMV due to the lap of inputs to the NA and DMV. The IML mainly receives projection from the brainstem and hypothalamus (Loewy, 1981; Schwanzel-Fukuda et al., 1984). The VLM, LC, and ventral raphe nuclei, which are located in the brainstem, give out straightforward projections to the IML (Amendt et al., 1979; Jones and Yang, 1985). Furthermore, the dorsomedial hypothalamus (DMH) and PAG are the convergence area where the projections from the sympathetic regulating brain areas are received (Guyenet, 2006; Fontes et al., 2011). Interestingly, the projections from the PVH, LHA, and posterior hypothalamus (PH), which are located in the hypothalamus, can directly reach the thoracic IML, and these projections are conserved in many species (Saper et al., 1976). Therefore, these nuclei mentioned above are the vital infracortical area, which are relative to the peripheral ANS and anxiety, and integrate affective and cognitive processes with CVD. The following section will focus on the anatomy and functional connection between the anxiety-related nuclei and cardiac ANS.

### Association Between Amygdala and Cardiac ANS

Circuit mapping in rodents indicates that projections from the medial amygdala (MeA) mainly liberate GABA to innervate the mPOA, PH, and BNST (Myers et al., 2014; Myers et al., 2016). Meanwhile, different subregions of the amygdala, including the lateral, basolateral, basomedial, and cortical parts, innervate the BNST through glutamatergic projection. (Myers et al., 2014). Notably, a mass of interconnections between the CeA and other regions of the amygdala can be observed (Davis, 1997; Davis et al., 2010). The CeA can reach up to the BNST, mPOA, and DMH through the GABAergic projections and affect many subregions of the brainstem, including the PB, LC, NTS, raphe, and rostral VLM (Hermann et al., 1997; Prewitt and Herman, 1998; Saha, 2005; Myers et al., 2014). Therefore, the amygdala is considered crucial nuclei to regulate the response to stress through GABA-GABA synaptic connections (Prewitt and Herman, 1998; Myers et al., 2012; Myers et al., 2014; Russell and Shipston, 2015).

Ample animal research has investigated the function and mechanism of the amygdala in the regulation of cardiac ANS. From electrolytic lesion or electrochemical stimulation studies, CeA likely participates in the modulation of BP reaction to stress (Saha, 2005). For instance, electrolytic lesions of the CeA in borderline hypertensive rats could attenuate pressor responses to stress (Sanders et al., 1994). In addition, other studies also suggested that the CeA is essential for conditioned cardiovascular responses to different types of stress (LeDoux et al., 1988; Phelps and LeDoux, 2005; Wilensky et al., 2006). In contrast, local injection of cobalt chloride into the MeA led to alterations in HR during acute restraint stress though the mean arterial pressure (MAP) displayed no change (Fortaleza et al., 2009). The results from other studies indicated that during stress alterations of HR and MAP were respectively regulated by the noradrenergic system and histaminergic system in the MeA (Fortaleza et al., 2012a; Fortaleza et al., 2012b; de Almeida et al., 2015). Without stress, BLA also contributes to an increase in HR and MAP after local injection of GABA receptor antagonists through the cell signal pathway related to the NMDA and AMPA (Sajdyk and Shekhar, 1997; Soltis et al., 1997). Furthermore, angiotensin-II metabolite binding to the Mas receptor in the BLA can lead to the reduction of MAP and HR responses to air-jet stress (Oscar et al., 2015).

### Association Between BNST and Cardiac ANS

The BNST is characterized by its connections with hypothalamic and brainstem nuclei, which are intimately correlated with cardiac ANS (Dong et al., 2001a; Dong and Swanson, 2004a; Dong and Swanson, 2004b; Spencer et al., 2005). The BNST is considered crucial in regulating physiological functions including an autonomic, neuroendocrine, and behavioral response (Ulrich-Lai and Herman, 2009; Davis et al., 2010). The results from previous investigations indicated a mutual interconnection between the BNST and centro-MeA and the projection from the HC and mPFC to the BNST (Shammah-Lagnado et al., 2000; Dong et al., 2001b; Herman et al., 2003; Vertes, 2004; Spencer et al., 2005; Radley et al., 2009). The BNST provides direct input to the DMV, which displayed the projection from the anterolateral and rhomboid divisions of the BNST to the DMV (Dong and Swanson, 2003; Dong and Swanson, 2004a). In addition, the NA also receives afferent input from the rhomboid BNST (Dong and Swanson, 2003).

Rodent studies also proved a modulatory role for the BNST in the regulation of cardiac ANS to stress (Crestani et al., 2013). The BNST was considered a vital part of regulating alterations to the cardiovascular system during emotional stress. Another study also found that the behavioral alterations induced by local stimulation of the BNST were akin to those arising from restraint stress, which indicated the involvement of the BNST in response to stress (Casada and Dafny, 1993). Furthermore, inhibition of local neurotransmission in the BNST can lead to the elevation of HR during acute restraint stress though the increase of BP was not significant (Crestani et al., 2009). In contrast, research using a conditioned stress model indicated a different result (Resstel et al., 2008).

During contextual fear conditioning, the freezing behavior and elevation of BP and HR were mitigated after ablation of the BNST (Resstel et al., 2008). Accordingly, different types of emotional stress can affect the degree of regulation by the BNST on the cardiac ANS.

### Association Between mPFC and Cardiac ANS

The multi-synaptic projections from the CNS to sympathetic neurons arise not only from the amygdala but also from the IL-mPFC, which has been revealed by injecting pseudorabies virus (a kind of trans-neuronal retrograde tracer) into the stellate ganglion (sympathetic neurons regulating the heart) or the adrenal gland (Westerhaus and Loewy, 2001).

The IL-mPFC plays an important role in regulating the stress responses of the cardiac ANS. On the one hand, this area can innervate the NTS through glutamatergic projection, whereas on the other hand, stress can activate the GABAergic cells of the PH, which originated from the IL-mPFC (Vertes, 2004; Myers et al., 2014; Herman et al., 2016; Myers et al., 2016). In addition, the GABAergic neurons in the anterior BNST and the rostral part of the raphe both received projection from the PL-mPFC (Vertes, 2004; Radley et al., 2009).

Previous studies indicated that the IL-mPFC and PL-mPFC subregions in rats may differentially regulate the cardiovascular stress response. The mPFC is important in the buildup to a stress response that relies on experienced outcomes. For instance, the responses of HR and MAP can be attenuated by injection of cobalt chloride inclusively into PL-mPFC and IL-mPFC even under the contextual fear conditioning (Resstel et al., 2006). The studies, investigating the association between stress responses and auditory-cued fear conditioning, found that the lesion location in the mPFC determined the difference in HR response to the conditioned stimulation (Frysztak and Neafsey, 1994). Especially, lesions of the entire mPFC by suction needle could lessen HR responses to stress. However, tachycardia responses elevated more significantly after specific aspiration in the dorsal mPFC (including the PL), which indicated this area can inhibit the sympathetic nervous system (Frysztak and Neafsey, 1994). Sympathetic mediated tachycardia can be lessened after local injection of excitotoxic chemical agents into the ventral-mPFC (including the IL) to destruct the normal function of this area, which indicated cardiovascular responses to learned fear depend on mediation from different subregions of the mPFC (Frysztak and Neafsey, 1994). The local injection of cobalt chloride into the PL could induce increased tachycardia responses to acute restraint stress without alteration of the MAP. On the contrary, the neurotransmission in IL displayed the opposite function, as the promotion of stress-induced tachycardia could be inhibited by local injection of cobalt chloride into the IL (Tavares et al., 2009). Furthermore, another study found that the responses of the HR or MAP to other stressors, including cage change, restraint, and air-jet, received no impact after inhibition of IL with muscimol (a kind of GABA agonist) (Müller-Ribeiro et al., 2012). Nevertheless, the results from this same research indicated that the NMDA-mediated signal activation in IL could recede the HR and MAP response during air-jet stress. Collectively, these results indicated that the cardiovascular responses to stress can be inhibited by the PL-mPFC, whereas IL-mPFC possessed an opposite function to induce sympathetic activation.

### Association Between HC and Cardiac ANS

The neurons of the sympathetic nervous system received multi-synaptic projections from the ventral part of the HC (Westerhaus and Loewy, 2001). The neurons in the ventral subiculum part of the HC comprises the primary stress regulation area of the HC, some of which provide efferents to neurons in the anterior part of the BNST which also receive projection from the PL (Radley and Sawchenko, 2011).

In addition, the ventral subregion of the HC innervates the LHA, mPOA, and medulla through projection (Köhler, 1990; Myers et al., 2014).

Furthermore, HC may regulate cardiac ANS. In rodents, local stimulation of the HC with electricity or chemical agents induces the reduction of the HR and MAP (Ruit and Neafsey, 1988). Importantly, after local stimulation of the ventral but not dorsal part of the HC, alterations of the cardiovascular system can be prevented by mPFC lesions (Ruit and Neafsey, 1988). To summarize, these results indicate that the neural circuit of the ventral hippocampal-mPFC modulates the suppression of HR and MAP, but stimulating the dorsal subregion of hippocampal inhibits HR and MAP through a disparate pathway. The results from another study indicated that cardiovascular responses to restraint stress can be enhanced through activation of the NMDA receptor in the dorsal HC, which differed from the consequence of stimulating the dorsal hippocampal without stress (Ruit and Neafsey, 1988; Moraes-Neto et al., 2014).

### Autonomic Neural Circuit May Contribute to CVD

The nuclei mentioned above may constitute a neural circuit, which can detect and interpret potential threats and finally induce anxiety and physiological response. In this regard, sensory information may be transmitted both forward (amygdala-BNST-HC-mPFC-downstream effector nuclei) and backward (mPFC/HC-amygdala-BNST) in this macrocircuit (Calhoon and Tye, 2015). The central neural interconnection, which consists of the mPFC, amygdala, and HC, adjusts emotions and awareness (Drevets, 1999; Drevets, 2000; Padilla-Coreano et al., 2016), and projections from these areas converge on important intracortical locations, primarily including the BNST, different subregions of the hypothalamus (e.g., LHA, mPOA, and PH) and distinct regions of the brainstem (e.g., VLM, raphe nuclei, and NTS) (Herman et al., 2003). This organization permits the transmission of limbic information downstream, which directly project into sympathetic and parasympathetic preganglionic neurons. This autonomic neural circuit of intracortical subregions constitutes the multi-synaptic central neural network that links emotional processes to physiological activity (Dampney, 2015). The complex interconnections mentioned above were mainly confirmed in various rodent studies; however, research on non-human primates also showed highly similar descending pathways in their ANS (Dum et al., 2016).

Accordingly, ANS may act as the pathway linking anxiety and CVD. Dysregulation of ANS especially sympathetic nervous system overactivity can contribute to cardiovascular pathology, including ischemic heart disease, hypertension, arrhythmias, and HF, and even contribute to fatal outcomes (Bairey Merz et al., 2015). In addition, a recent meta-analysis (including 2,834 patients) has found that patients with anxiety disorder exhibited significant reductions in HRV compared to controls, indicating an imbalance between the sympathetic and parasympathetic nervous systems, while ANS shifted toward sympathetic nervous system hyperactivity (Alvares et al., 2016). Therefore, modulating the autonomic neural circuit through targeting the anxiety-related nuclei may act as a treatment for anxiety comorbidities in CVD.

## Potential Neuromodulation for Treating Comorbidity Anxiety With CVD

Effective treatment for alleviating anxiety comorbidity with CVD is scarce. Existing common strategies for patients with CVD and anxiety include cognitive-behavioral therapy (CBT), medication, and a combination of both. However, the effect of these strategies is mixed and seldom improves both CVD and anxiety (Celano et al., 2018). Therefore, it is of great significance to search for other potential concomitant treatments for anxiety and CVD, such as transcranial magnetic stimulation (TMS) or transcranial focal ultrasound stimulation (tFUS). These approaches directly affect certain nuclei or brain regions to rebalance the autonomic neural circuit for the regulation of anxiety and the cardiovascular system (Calhoon and Tye, 2015). This part mainly focuses on several brain neuromodulations, including vagal nerve stimulation (VNS), TMS, and tFUS, which are probable potential treatments for psychological and psychiatric diseases by affecting ANS (Temel et al., 2012; Kubanek, 2018).

### Vagal Nerve Stimulation

VNS possesses bidirectional effects on the central and peripheral nervous systems to modulate the brain activity and cardiovascular function (Rossi et al., 20162067). However, traditional VNS is invasive and incurs several intra-operative risks, including infection, demolishment of the vagus nerve, trachyphonia, polypnea, and re-intervention to replace the exhausted battery (Fahy, 2010; Kamath et al., 2010; Spuck et al., 2010).

A newly non-invasive method, called transcutaneous VNS (tVNS), has been investigated, and this new neural modulation overcomes the disadvantages mentioned above as well as permits patient-administered stimulation on demand (Ben-Menachem et al., 2015). Researchers have demonstrated that this technique can alleviate chronic pain disorders and modulate the default mode network in major depressive disorder patients (Napadow et al., 2012; Fang et al., 2016). Damon et al. found that tVNS can improve the ANS response to emotional startle in patients with PTSD through elevating vagal tone and reducing sympathetic activation (Lamb et al., 2017). In addition, compared with the sham group, tVNS decreased the sympathetic tone and regulated the parasympathetic/sympathetic function in healthy volunteers after traumatic stress, which displayed the elevation of the pre-ejection period (PEP) of the heart (an indicator for cardiac sympathetic activity) and the photoplethysmogram (PPG) amplitude (a marker for peripheral sympathetic function) (Gurel et al., 2020). Furthermore, VNS could be a practical solution to rebalance the ANS, which is applied for the treatment of HF, AF, and CHD (Shinlapawittayatorn et al., 2013; Premchand et al., 2014; Chen et al., 2015). A recent proof-of-concept study has confirmed that low-level tragus stimulation can reduce myocardial ischemia-reperfusion injury in patients with acute MI and proposed the possibility that this non-invasive strategy may be used to treat patients with ST-segment elevation MI undergoing primary PCI (Yu et al., 2017). Accordingly, the VNS treatment strategy may be a promising approach to remit patients with anxiety and CVD.

### Transcranial Magnetic Stimulation

TMS, a newly non-intrusive neural modulatory strategy, can affect the activity of the cerebral cortex through currents produced by a coil that is located on the scalp (Rossini and Rossi, 2007). High-frequency (≥5 Hz) TMS increases cortical excitability, whereas low-frequency (≤1 Hz) TMS reduces cortical excitability (Rossi et al., 2009). The suggestions from a recent guideline indicate that TMS can be considered an effective treatment to alleviate chronic pain syndromes, medication-resistant depression, and negative symptoms of schizophrenia (Lefaucheur et al., 2020). The mild elevation of parasympathetic tone in healthy subjects, which displayed significant bradycardia, can be induced by local stimulation on the right hemisphere by low-frequency TMS, but no alteration of the sympathetic drive was observed (Gulli et al., 2013). In patients in a vegetative state, high-frequency TMS-stimulating M1 (principal brain areas involved in motor function) transiently induced the increase of HR, which indicated that local stimulation of M1 can regulate the function of ANS under the circumstance of no motor response (Manganotti et al., 2013). Furthermore, a study (including 52 patients with depression) found that compared with treatment with the serotonergic agent, high-frequency TMS stimulation of the left dorsolateral prefrontal cortex (DLPFC) daily for 2 weeks can rebalance the ANS, which displayed a decrease in the sympathetic/parasympathetic ratio through the analysis of HRV (Udupa et al., 2007).

Another study found that TMS can improve the scores of the clinician-administered PTSD scale in patients with PTSD after brief exposure to traumatic events. TMS also attenuated HR response to brief imaginal traumatic exposure, which indicated that the appliance of TMS may alleviate PTSD symptoms and regulate the ANS (Isserles et al., 2013). Although no direct evidence confirms the effect of TMS on CVD, the regulation of ANS through TMS may also indicate a potential ability to improve anxiety and CVD.

### Transcranial Direct Current Stimulation

Cortex excitability can be altered by transcranial direct current stimulation (tDCS) by the transmission of a transcranial constant electrical field to affect the course of cell membrane polarization (Priori et al., 1998; Paulus, 2003). The anode part of tDCS can elevate cortex excitability via transmitting the current to attract negative ions using electrodes located on the tissue surface, subsequently lowering the resting potential of the cell membrane threshold. On the contrary, cathodal tDCS can also impose the effect on the surroundings by attracting a positive charge, leading to the threshold elevation and reduction of cortical excitability. This technique raised the possibility of targeting the cortex and brainstem related to ANS, which contributes to our understanding of the interaction between the CNS and cardiovascular system as well as advances the treatment strategy for these pathological conditions (Parazzini et al., 2014). Despite the promising of this technique as a feasible treatment strategy for humans, only a few investigations have explored the effect of tDCS in regulating the cardiovascular function in humans (Vandermeeren et al., 2010; Brunoni et al., 2013; Schestatsky et al., 2013). M1 and DLPFC were the most common target for tDCS in most studies, and the temporal cortex was also chosen as a target to investigate the effect of local stimulation by tDCS on the autonomic nervous function (Montenegro et al., 2011). The results from previous research indicated that positive alterations of ANS induced by tCDS were mainly relevant to anodal stimulation, while another two studies found that the local stimulation on DLPFC or M1 by cathodal tDCS can also involve alterations of vasomotor reactivity (Beeli et al., 2008; Vernieri et al., 2010). Moreover, two studies intended to explore the impact of tDCS on the brainstem. The results displayed that stimulation applied on M1 or frontal midline using tDCS scarcely affected the cardiovascular function, and objectives in the tDCS group or sham group both squinted towards a progressive increase of sympathetic tone along with time (Vandermeeren et al., 2010; Santarnecchi et al., 2014). In fact, Santarnecchi et al. showed that the spontaneous activity of the motor cortex alongside the time course was likely associated with changes in HRV and BP in the absence of tDCS, and this association could be enhanced through the application of anodal tDCS on the motor cortex. This result indicated that tDCS could be used as a potential technique to determine the causal connection between the activity of specific subregions of the cerebral cortex and the function of the peripheral ANS (Santarnecchi et al., 2014). However, Carnevali et al. (2020) found that tDCS can reduce the degree of anxiety and attenuate HR acceleration and activation of the sympathetic nervous system/withdrawal of the vagal nervous system in healthy volunteers after completing the psychological stress test. In addition, local stimulation on the left DLPFC can decrease the number of faults for inconsistent stimuli and lower HR under a Stroop test in healthy participants (Angius et al., 2019). Although the current research related to the tDCS cannot determine the exact effect of tDCS on regulating the function of ANS due to the contradictory results from investigations and the diverse parameters of tDCS, this technique may potentially assist in alleviating anxiety comorbidity with CVD.

### Transcranial Focused Ultrasound

Transcranial focused ultrasound (FUS) is an emerging device for non-invasive neuromodulation that propagates low-intensity ultrasound through the skull and tissue to modulate regional brain activity (Tyler, 2011). FUS can act on target brain nuclei via two main mechanisms—thermal effect and mechanical effect (Kubanek, 2018). Compared with TMS, FUS performs better in an area with sufficiently tight focus and specific circuits deep in the brain (Fini and Tyler, 2017). A randomized, placebo-controlled, double-blind study has demonstrated the impact of FUS on right inferior frontal gyrus (rIFG) in healthy participants to modulate mood and emotional state (Sanguinetti et al., 2020). However, the average HR, HRV, and respiratory sinus arrhythmia (RSA) of these participants did not change after stimulation by FUS (Sanguinetti et al., 2020). On the contrary, the result from research into spontaneously hypertensive rats indicated that the HR and SBP can be significantly reduced after continuous FUS for 1 week (Li et al., 2020). These results indicate the potential role of FUS in emotion regulation. However, the contradictory cardiovascular response may be due to specific stimulation parameters of FUS required for exciting or inhibiting cellular activity (Plaksin et al., 2016). Moreover, a recently published study displayed that FUS can significantly improve the primary outcome in patients with treatment-refractory generalized anxiety disorder, as measured by the Hamilton Anxiety Inventory, and indicated FUS as a concomitant treatment with anxiety (Mahdavi et al., 2021). However, only limited evidence to date has implied the ability of FUS to improve the symptoms of anxiety and regulate cardiac ANS in patients with anxiety disorder accompanied by CVD or the risk thereof. Nevertheless, FUS can offer a potential direction to investigate concomitant treatment for anxiety comorbidity with CVD.

## Conclusion

**FIGURE 1:**
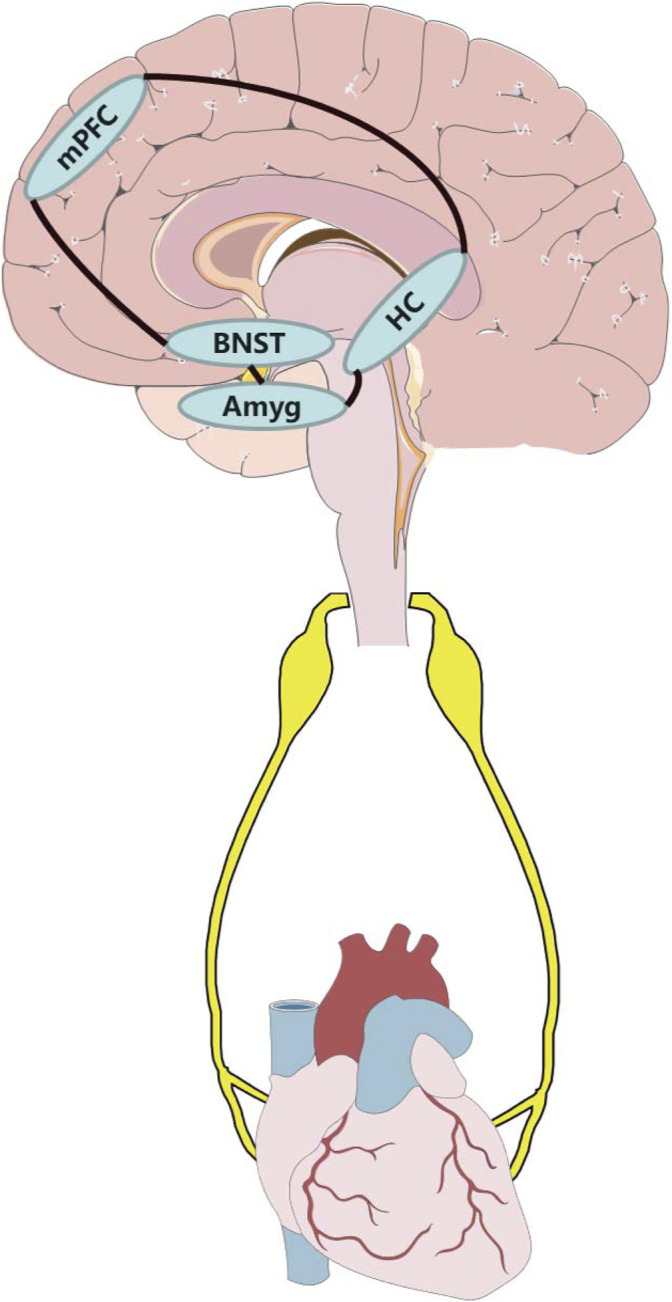
Potential anxiety-related neural circuit influence cardiovascular system through autonomic nervous system. Amyg, amygdala; BNST, bed nucleus of the stria terminalis; HC, hippocampus; mPFC, medial prefrontal cortex.

A large number of research studies have demonstrated the relationship between anxiety and CVD, yet the exact role of nuclei and the neural circuit responsible for anxiety remains unclear in CVD. This severely impedes progress in prevention and treatment for comorbidity anxiety with CVD. Therefore, further investigating the causal connection between alteration of anxiety neural nuclei and CVD is important for an in-depth understanding of the mechanism of coexisting psychiatric disease and CVD. Specifically, the autonomic neural circuit consisting of nuclei related to anxiety and ANS is of significant research interest for treating and preventing anxiety and CVD (Figure 1). Furthermore, the emerging neural modulation techniques may provide effective strategies to alleviate both anxiety and CVD through assisting traditional treatments (CBT and medication). However, exact parameters for these devices need to be established in further clinical trials to ensure safety and effectiveness for patients.

## Glossary

adBNST: anterodorsal BNST
AF: atrial fibrillation
ANS: autonomic nervous system
BLA: basolateral amygdala
BNST: bed nucleus of stria terminalis
BPV: blood pressure variability
CABG: coronary artery bypass graft surgery
CBT: cognitive-behavioral therapy
CeA: central nucleus of the amygdala
CHD: coronary artery disease
CNS: central nervous system
CVDs: cardiovascular diseases
DLPFC: dorsolateral prefrontal cortex
DMH: dorsomedial hypothalamus
DMV: dorsal motor nucleus of the vagus
fMRI: functional magnetic resonance imaging
FUS: transcranial focused ultrasound
GMD: gray matter density
HC: hippocampus
HF: heart failure
HRV: heart rate variability
IL: infralimbicinfralimbic mPFC
LC: locus coeruleus
LHA: lateral hypothalamic area
MACE: major adverse cardiovascular events
MAP: mean arterial pressure
MeA: medial amygdala
MI: myocardial infarction
mPFC: medial prefrontal cortex
mPOA: medial preoptic area
NA: nucleus ambiguus
NTS: nucleus of the solitary tract
OR: odds ratio
ovBNST: oval BNST
PAG: periaqueductal gray
PB: parabrachial nuclei
PCI: percutaneous coronary intervention
PEP: pre-ejection period
PH: posterior hypothalamus
PL: prelimbic mPFC
PPG: photoplethysmogram
PTSD: post-traumatic stress disorder
PVH: paraventricular hypothalamus
rIFG: right inferior frontal gyrus
rmPFC: rostro-mPFC
RSA: respiratory sinus arrhythmia
RSFC: resting-state functional connectivity
tDCS: transcranial direct current stimulation
tFUS: transcranial focal ultrasound stimulation
TMS: transcranial magnetic stimulation
tVNS: transcutaneous VNS
VLM: ventrolateral medulla
vmPFC: ventro-mPFC
VNS: vagal nerve stimulation
